# Factor Structure and Validity of the Japanese Version of the Emotional Availability Self-Report

**DOI:** 10.3390/children12070867

**Published:** 2025-06-30

**Authors:** Nozomi Kanehira, Young Ae Kang, Eriko Suwa, Sugako Asaeda, Toshihiko Tsutsumi, Keiko Tsuji, Koudai Fukudome, Mina Nakano, Masamichi Yuzawa

**Affiliations:** 1 Department of Psychology, Fukuyama University, 985-1 Sanzo, Higashimura-cho, Fukuyama-shi 729-0292, Hiroshima, Japan; mnakano@fukuyama-u.ac.jp; 2 Graduate School of Humanities and Social Sciences, Hiroshima University, 1-3-2 Kagamiyama, Higashi-Hiroshima-shi 739-8511, Hiroshima, Japan; 3 Clinic Ogura, 3-16-11 Wakabayashi, Setagaya-ku 154-0023, Tokyo, Japan; 4 Department of Psychology, Mejiro University, 4-31-1 Naka-Ochiai, Shinjuku-ku 161-8539, Tokyo, Japan; 5 Hiroshima City Mental Health and Welfare Center, 11-27 Fujimi-cho, Naka-ku, Hiroshima-shi 730-0043, Hiroshima, Japan; 6 Department of Psychology, Osaka University of Human Sciences, 1-4-1 Shojaku, Settsu-shi 566-8501, Osaka, Japan; 7 School Counselor, Hiroshima Prefectural Board of Education, 9-42 Motomachi, Naka-ku, Hiroshima-shi 730-0011, Hiroshima, Japan; 8 Department of Contemporary Human Sciences, St. Catherine University, 660 Hojo, Matsuyama-shi 799-2496, Ehime, Japan; fukudome-k@catherine.ac.jp

**Keywords:** Emotional Availability Self-Report, mothers of infants and toddlers, reliability, validity, Japan

## Abstract

Background/Objectives: Emotional interactions between mothers and children are essential for later developmental outcomes such as children’s health, social competence, and language skills. However, an observational assessment of such interactions cannot reveal how mothers perceive their relationships with their children. Therefore, in this study, we translated and validated the Japanese version of the Emotional Availability Self-Report (EA-SR-J) and examined its factor structure, reliability, and validity. Methods: The participants were 739 Japanese mothers with children aged from 1 month to 5 years and 11 months. Results: Exploratory factor analysis revealed 19 items and four factors (Affect Quality, Hostility, Mutual Attunement, and Child Involvement with Parent) for the EA-SR-J. However, Intrusiveness was removed, and some items were assigned to different factors compared with the original scale. Cronbach’s alpha of each subscale ranged from 0.81 to 0.88, indicating sufficient internal consistency. The convergent validity of the scale was confirmed with expected correlations with variables related to attachment and child-rearing style. Discriminant validity was confirmed by group differences in variables related to children’s autism spectrum disorder symptoms and mothers’ depression symptoms. Conclusions: Although the EA-SR-J contains fewer items than the original scale, with the Intrusiveness item removed, it remains a reliable and valid tool. Further studies using the Emotional Availability Scale based on observation are needed in the future.

## 1. Introduction

Emotional availability (EA) refers to the ability of two people to form an emotional connection with each other [1]. It is grounded in attachment theory [2] and Emde’s emotion framework [3], expanding these foundations to emphasize not only survival behaviors but also the quality of “emotional” and “dyadic” interactions [4]. Specifically, EA in the mother–child relationship refers to the mother’s capacity to appropriately identify and respond to her child’s emotional needs and cues, including the child’s capacity to elicit the mother’s emotional responses. Secure emotional interactions between mothers and children have been linked to positive developmental outcomes in children, including health, social competence, and language skills [5,6,7].

Numerous studies on EA have been conducted thus far, and in recent years, EA has been examined not only among mothers and children with typical development, but also in at-risk clinical populations, such as children with disabilities and parents with mental illness. These studies suggest that EA is likely to be inhibited by various risk factors in both mothers and children, such as in children with autism spectrum disorder (ASD) and mothers with depressive symptoms [1]. Thus, there is a growing need for accessible and culturally appropriate EA assessment tools, particularly for early detection and intervention in clinical populations.

The EA System measures EA. The Emotional Availability Scale (EAS) is based on the observations made by certified or accredited individuals, and objectively measures the quality of mutual influence between adults and children along six dimensions (adult: Sensitivity, Structuring, Non-Intrusiveness, Non-Hostility; child: Responsiveness to the Adult, Involvement of the Adult) [4]. With over 400 studies conducted in 25 countries, the EAS is well established as an observational measure of adult–child relationships [8]. However, an observational assessment of EA does not reveal how mothers perceive their relationships with their children.

Therefore, the Emotional Availability Self-Report (EA-SR), a self-report questionnaire completed by caregivers (adults involved in the care of children), was developed [9,10]. It comprises five subscales with items based on the EAS rating scale. The subscales are “Hostility” and “Intrusiveness,” which refer to the caregivers’ contribution to the relationship as perceived by the caregiver; “Child Involvement with Parent (Child Involvement),” which refers to the child’s contribution to the relationship as perceived by the caregiver; and “Mutual Attunement” and “Affect Quality,” which refer to dyadic items, that is, items for which both children and caregivers contribute to the relationship [10]. Although the EA-SR is moderately correlated with the EAS, Vliegen et al. (2009) [10] stated that differences between the observer-rated EAS and the mother’s self-reported EA-SR provide vital information. By using both scales and carefully understanding their differences, it is possible to identify the differences between professional objective assessments and mothers’ subjective perceptions. Notably, the EA-SR has been used to measure the effectiveness of EA interventions (e.g., [11,12]).

However, the EA-SR has been less extensively studied than the EAS, and the internal consistency of its subscales reported in prior studies varies widely (0.42–0.87; [13,14]). In particular, the Intrusiveness factor has not been shown to correlate with any subscale of the EAS by observation [10]. Therefore, it would be valuable to accumulate more research data on the factor structure. In addition, no Japanese version of the EA-SR exists to date. Most studies using the EA-SR have focused on Western cultures. Nonetheless, the characteristics of mother–child interactions require further investigation, as they likely exhibit qualitatively different characteristics across different cultures. Research on the EA-SR in Eastern cultures, particularly in Japan, emphasizing relational and mutual cooperation, may provide new insights into mother–infant relationships by capturing a maternal perspective that differs from that in individualistic and independence-oriented Western cultures.

With Biringen’s permission, we translated and validated the Japanese version of the EA-SR (EA-SR-J) and examined its factor structure, reliability, and validity. The self-reported associations among the emotional bond between mother and infant, child’s attachment behavior, and the mother’s attachment and child-rearing styles were examined by calculating Pearson’s correlation coefficients to confirm the convergent validity of the EA-SR-J.

EA is based on attachment theory, and the association between EA and attachment has been tested in many previous studies (e.g., [15,16,17]). These studies found that secure attachment is associated with maternal sensitivity and responsiveness. Therefore, in this study, we hypothesized that the positive subscales of child attachment behavior would be negatively correlated with Hostility and Intrusiveness, and positively correlated with Child Involvement, Affect Quality, and Mutual Attunement in the EA-SR-J. Negative subscales of child attachment behavior, emotional bonding between mother and infant, and the mother’s attachment style were expected to show inverse correlations. A responsive child-rearing style in which mothers use loving expressions and try to fulfill their children’s desires is thought to enhance the quality of interaction. Therefore, we predicted that a responsive child-rearing style would show negative correlations with Hostility and Intrusiveness, and positive correlations with Child Involvement, Affect Quality, and Mutual Attunement in the EA-SR-J. By contrast, because the controlling child-rearing style that mothers adopt regardless of their children’s intentions is a concept similar to Intrusiveness in the EA-SR-J, we predicted a positive correlation with this subscale.

To determine the discriminant validity of the EA-SR-J, we compared the mean values of its subscales for children with and without ASD symptoms and mothers with and without depressive symptoms. Based on Gul et al. and Van IJzendoorn et al. [18,19], we predicted that the group with ASD symptoms (ASD group) would have significantly lower EA-SR-J Child Involvement, Affect Quality, and Mutual Attunement than the group without ASD symptoms (non-ASD group). Furthermore, based on Vliegen et al. and Frigerio et al. [10,20], we expected that the group with depression symptoms (depression group) would have significantly lower EA-SR-J Child Involvement, Affect Quality, and Mutual Attunement and substantially higher Hostility and Intrusiveness than the group without depression symptoms (non-depression group).

## 2. Materials and Methods

### 2.1. Survey Procedures and Participants

This study was conducted throughout Japan via an online survey of mothers with children aged from one month to five years and 11 months, who were registered as research monitors at a marketing research company (Cross Marketing Inc., Shinjuku-ku, Japan). Children’s ages were divided into four categories (1–11 months, 12–17 months, 18–26 months, and 27–71 months) according to the age limits of the scale used for the validity study. The basic sampling criteria targeted women aged 20–49 years who had at least one child in the specified age range. In cases where a mother had multiple preschool-aged children, one child was selected for the study. This child was assigned to one of the four age categories, with priority given to age groups with fewer participants to balance the sample distribution. This study included 739 participants (*M*_age_ = 35.36 years, *SD* = 6.44) with a child (372 boys, 367 girls; *M*_age_ = 25.75 months, *SD* = 18.35; 224 were 1–11 months old, 21 were 12–17 months old, 243 were 18–26 months old, and 251 were 27–71 months old).

### 2.2. Measures

#### 2.2.1. EA-SR-J

Permission to translate and validate the EA-SR-J was obtained from Biringen, the author and the copyright owner of the EA-SR. First, the first through sixth authors (four of whom were certified or accredited to use the EA) translated the original scale into Japanese and engaged in discussions to determine the appropriate translation. The provisional Japanese version was back translated by a translation company (Crimson Interactive, Tokyo, Japan). Finally, the back-translation results were sent to the original author, who confirmed that there were no discrepancies in meaning between the original and back-translated scales. The EA-SR-J comprises 36 items and five subscales: Mutual Attunement (10 items; e.g., “I find it hard to attune myself to the rhythm of interaction with this particular child” [reverse-coded]); Affect Quality (five items; e.g., “I like to have eye-contact with my child”); Intrusiveness (six items; e.g., “I always want to know what my child is up to or is thinking”); Hostility (six items; e.g., “It happens that I react in an angry way to my child”); and Child Involvement with Parent (nine items; e.g., “My child calls me to come and play, talk, and/or interact”). Each item is rated on a five-point Likert-type scale ranging from 0 (do not agree at all) to 4 (totally agree). The higher the score, the higher the expression of the characteristics of each subscale.

#### 2.2.2. Demographics

The demographic data of the participants are presented in Table 1. Participants were also asked about their area of residence, age, and spouse’s occupation. The following scales were selected for the validity of the EA-SR-J (2.2.3–2.2.8): for convergent validity, we used self-report measures of the emotional bond between mothers and infants, children’s attachment behavior, and mothers’ attachment and child-rearing styles. For discriminant validity, we used children’s ASD tendencies and mothers’ depression tendency scales. The target age varied for each scale.

#### 2.2.3. Postpartum Bonding Questionnaire—Japanese Version of the 14-Item Scale

Only mothers of children aged 1–11 months (*n* = 224) were asked to respond to the Postpartum Bonding Questionnaire—Japanese Version of the 14-Item Scale (PBQ-J-14), which measures the emotional bond between the mother and infant [21]. The 14 items are rated on a six-point Likert scale ranging from 0 (not at all) to 5 (always). For example, one item reads “I wish I could go back to the time before I had my baby.” Higher scores indicated that the emotional bond between the mother and child is problematic. The reliability coefficient for this study was 0.86.

#### 2.2.4. Attachment Behavior Checklist

Only mothers of children aged 12–71 months (*n* = 515) were asked to respond to the Attachment Behavior Checklist (ABCL), which measures children’s attachment behaviors [22]. This scale has three subscales: Understanding of Caregiver’s Mind (seven items; α = 0.80; example item: “When you ask your child to do something, they quickly understand what you want”); Dysregulation of Emotions (nine items; α = 0.82; example item: “Your child easily gets angry at you”); and Secure Base (four items; α = 0.69; example item: “Even while playing, your child knows where you are, calls out to you, or notices when you move”). The 20 items are rated on a five-point Likert scale ranging from 1 (does not apply at all) to 5 (applies very much). Higher scores indicate a higher tendency toward the behaviors assessed by each subscale.

#### 2.2.5. Experiences in Close Relationships Inventory—The Generalized Other Version

Mothers of children aged 1–71 months (*n* = 739) were asked to respond to the Experiences in Close Relationships Inventory—Generalized Other (ECR-GO) version, which assesses mothers’ attachment styles [23]. This scale has two subscales: Anxiety (18 items; α = 0.92; example item: “I worry that others will abandon me”) and Avoidance (12 items; α = 0.81; example item: “I try not to get too close to other people”). The 30 items are rated on a seven-point Likert scale ranging from 1 (does not apply at all) to 5 (applies very well). Higher scores indicate more attachment style problems.

#### 2.2.6. Maternal or Paternal Child-Rearing Style Scale

Only mothers of children aged 18–71 months (*n* = 494) were asked to respond to the Child-Rearing Style Scale (Child-Rearing Style), which was used to measure mothers’ attitudes toward child rearing [24]. This scale has two subscales: Responsiveness (eight items; α = 0.76; example item: “I show affection by hugging my child or speaking gently”) and Control (eight items; α = 0.62; example item: “When my child doesn’t do what they are supposed to, I tell them to do it”). Although the internal consistency for the Control subscale was somewhat modest (α = 0.62), this subscale has been psychometrically validated in previous research. In the present study, we retained the original structure without modification to preserve the scale’s theoretical and empirical integrity. The 16 items are rated on a four-point Likert scale ranging from 1 (does not apply at all) to 4 (applies perfectly). Higher scores indicate a higher tendency toward the behaviors assessed by each subscale.

#### 2.2.7. Modified Checklist for Autism in Toddlers

Only mothers of children aged 18–26 months (*n* = 243) were asked to respond to the Modified Checklist for Autism in Toddlers (M-CHAT), which measures ASD symptoms in children [25]. For example, one item reads, “Does your child show interest in other children?” A total of 23 items are rated on a two-point scale (0 = yes, 1 = no; α = 0.69). Higher scores indicate a higher tendency for ASD. A child is considered to have failed the M-CHAT if they fail three or more items on the checklist. Notably, the M-CHAT used in this study differed from the original Japanese version in that no pictures were used.

#### 2.2.8. Japanese Version of the Patient Health Questionnaire-9

Mothers of children aged 1–71 months (*n* = 739) were asked to respond to the Japanese version of the Patient Health Questionnaire-9, which was used to measure maternal depressive tendencies [26]. For example, one item reads, “I feel tired or have no energy.” The nine items are rated on a four-point Likert scale ranging from 0 (not at all) to 3 (almost every day) (α = 0.89). The higher the score, the higher the tendency for depression. Of the maximum score of 27, 0–4 points indicated no symptoms, 5–9 points indicated mild symptoms, 10–14 points indicated moderate symptoms, 15–19 points indicated moderate-to-severe symptoms, and 20–27 points indicated severe symptoms. When administering the survey, two trap items required respondents to provide specific answers to exclude those engaging in satisfaction.

### 2.3. Statistical Analysis

First, confirmatory factor analysis was conducted to examine whether the factor structure was similar to that of the original EA-SR. The sample was then randomly divided into two parts, and exploratory and confirmatory factor analyses were conducted to examine and refine the factor structure of the EA-SR-J. The goodness-of-fit criteria were set as CFI, TLI > 0.90, and RMSEA < 0.08 [27,28]. Cronbach’s alpha was used to confirm reliability. Pearson’s correlation coefficients were calculated between the EA-SR-J and PBQ-J-14 scores and the ABCL, ECR-GO, and Child-Rearing Style Scale scores to assess convergent validity. Finally, to determine the discriminant validity of the EA-SR-J, an unpaired *t*-test was conducted with EA-SR-J subscale scores as the dependent variable and the presence of ASD or maternal depressive symptoms as independent variables. SPSS Windows software version 27 and AMOS version 27 were used for statistical analyses.

## 3. Results

### 3.1. Confirmatory Factor Analysis on the Original Scale

Confirmatory factor analysis was conducted on data from 739 participants, assuming the factor structure of the original scale [10]. The results show that the model fit poorly (CFI = 0.712, TLI = 0.691, and RMSEA = 0.090). These results suggest that the factor pattern of the original scale does not apply to the EA-SR-J. Therefore, we randomly divided the 739 participants into two subsamples and reexamined the factor structure of the EA-SR-J using exploratory and confirmatory factor analyses. A subsample of 369 participants was used to conduct exploratory factor analysis (mothers: *M*_age_ = 34.42 years, *SD* = 6.5; children: 177 boys, 192 girls; *M*_age_ = 25.73 months, *SD* = 18.35; 112 were 1–11 months old, 10 were 12–17 months old, 122 were 18–26 months old, and 125 were 27–71 months old). The other subsample of 370 participants was used to conduct confirmatory factor analysis (mothers: *M*_age_ = 35.3 years, *SD* = 6.4; children: 195 boys, 175 girls; *M*_age_ = 25.76 months, *SD* = 18.4; 112 were 1–11 months old, 11 were 12–17 months old, 121 were 18–26 months old, and 126 were 27–71 months old).

### 3.2. Exploratory Factor Analysis

Exploratory factor analysis (maximum likelihood method with Promax rotation) was conducted on data from 369 EA-SR-J participants. Consequently, four factors were considered appropriate. Subsequently, items with low factor loadings (less than 0.40) and cross-loadings were excluded. Subsequently, 17 items were excluded and a four-factor structure of 19 items was identified.

### 3.3. Confirmatory Factor Analysis

Confirmatory factor analysis was conducted using a separate sample (370 participants) from the exploratory factor analysis. The results showed that CFI = 0.915, TLI = 0.901, and RMSEA = 0.071 met the goodness-of-fit criteria. Therefore, the EA-SR-J adopted the 19-item, four-factor structure. The factor pattern matrices are presented in Table 2.

The first factor with six items (e.g., “I like to have eye contact with my child”) showed a high loading on the Affect Quality items of the original scale. Similarly, the first factor in this study was named “Affect Quality” because it represented the emotional interaction between mothers and children (*M* = 3.27, *SD* = 0.59).

The second factor with four items (e.g., “It happens that I raise my voice to my child”) showed a high loading on the Hostility quality items of the original scale. Similarly, the second factor in this study was “Hostility,” because it represented hostility from mothers to children (*M* = 1.77, *SD* = 1.11).

The third factor with five items [e.g., “I find it hard to structure my child’s behaviors and actions when they do things that are not allowed or are dangerous (R)”] showed high loadings on the Mutual Attunement items of the original scale. Similarly, in this study, the third factor was named “Mutual Attunement” because it represented mothers and children attuned to each other’s intentions (*M* = 2.55, *SD* = 0.87).

The fourth factor with four items (e.g., “My child often asks me to look at what they are playing or doing”) showed a high loading on the Child Involvement with Parents items in the original scale. Similarly, in this study, the fourth factor was named “Child Involvement with Parents” because it represented the involvement behavior of children with the mothers (*M* = 3.02, *SD* = 0.81).

### 3.4. Internal Consistency of the EA-SR-J

Cronbach’s alpha was calculated for each of the two samples used in the analysis to examine the internal consistency of the EA-SR-J: Affect Quality (α = 0.81, 0.82), Hostility (α = 0.87, 0.88), Mutual Attunement (α = 0.81, 0.82), and Child Involvement (α = 0.81, 0.82). The internal consistency was confirmed to be sufficient.

### 3.5. Examining the Validity of the EA-SR-J

Pearson’s correlation coefficients were calculated between the scores of the EA-SR-J subscales and the PBQ-J-14, ABCL, ECR-GO, and Child-Rearing Styles Scale using data from 739 respondents to examine the convergent validity of the EA-SR-J (Table 3). The number of participants included in each analysis varied depending on the applicable age range for each validation scale: PBQ-J-14 (*n* = 224), ABCL (*n* = 515), ECR-GO (*n* = 739), and Child-Rearing Styles Scale (*n* = 494). Based on Ferguson’s recommended values for effect sizes [29], we used *r* = 0.2 as the criterion, which was considered the minimum substantially meaningful magnitude of the correlation coefficient.

The results generally supported our hypotheses. Specifically, Hostility was positively correlated with PBQ-J-14, Dysregulation of Emotions on the ABCL, and Anxiety on the ECR-GO. Hostility scores were also negatively correlated with Responsiveness in the Child-Rearing Styles Scale. Affect Quality and Child Involvement were positively correlated with Understanding the Caregiver’s Mind and Secure Base of the ABCL, including Responsiveness in the Child-Rearing Styles Scale. Affect Quality was negatively correlated with PBQ-J-14 scores. Mutual Attunement was negatively correlated with PBQ-J-14, Dysregulation of Emotions on the ABCL, and Anxiety on the ECR-GO. Mutual Attunement was also positively correlated with Responsiveness on the Child-Rearing Styles Scale. Thus, convergent validity was demonstrated for all the subscales.

As an additional exploratory analysis, unpaired *t*-tests were conducted to examine whether the EA-SR-J subscale scores differed by child gender (male vs. female), with each EA-SR-J subscale as the dependent variable and gender as the independent variable. No significant differences were found across any of the four subscales (all *p* > 0.10; e.g., Affect Quality: *t* (737) = 1.16, *p* = 0.25). Similarly, one-way ANOVAs were conducted to examine whether EA-SR-J subscale scores differed by birth order (first-born to fourth-born), with each subscale as the dependent variable and birth order as the independent variable. No significant differences were found across any of the four subscales (all *p* > 0.10; e.g., Hostility: *F* (3, 735) = 1.66, *p* = 0.18). In contrast, one-way ANOVAs were conducted to examine differences across child age categories (1–11, 12–17, 18–26, and 27–71 months), with age group as the independent variable. Significant group differences were found in several subscales (e.g., Mutual Attunement: *F* (3, 735) = 11.86, *p* < 0.01). Specifically, scores for Mutual Attunement and Affect Quality tended to decrease with age, whereas scores for Hostility and Child Involvement tended to increase. These findings suggest the potential developmental influence on parental emotional availability.

To determine the discriminant validity of the EA-SR-J, an unpaired *t*-test was conducted with the EA-SR-J subscale scores as the dependent variable and ASD symptoms with or without maternal depressive symptoms as independent variables (Table 4).

The results generally supported our hypotheses. Specifically, the ASD group had significantly lower scores on Affect Quality, Mutual Attunement, and Child Involvement than the non-ASD group. Additionally, the depression group scored significantly higher on Hostility and considerably lower on Affect Quality, Mutual Attunement, and Child Involvement than the non-depression group. Thus, the results demonstrated the discriminant validity of the subscales.

## 4. Discussion

In this study, we translated and validated the EA-SR-J and examined its factor structure, reliability, and validity. The results revealed 19 items across four factors for the EA-SR-J, differing from the original scale. Of the four factors, although some items for Hostility and Child Involvement were deleted, the items were arranged in the same way as in the original scale and it was assumed that the same content as in the original factor was measured. In addition, the original scale items corresponding to the Affect Quality and Mutual Attunement factors had high factor loadings. However, some items from other factors were also included. For example, the Affect Quality factor included items from Intrusiveness (e.g., “When I’m at home together with my child, we interact and talk to each other a lot”). Affect Quality includes aspects such as the quality of the mother–child interaction. The Intrusiveness item included in Affect Quality in the EA-SR-J is also thought to have aspects of positive interaction. By examining the content of the items belonging to other factors in the original scale, we determined that they corresponded to the factor content of this study. Therefore, the four factors that supported the original scale were named in the EA-SR-J.

The internal consistency of the EA-SR-J ranged from 0.81 to 0.88, confirming sufficient internal consistency compared with previous research results (0.42–0.87 [13,14]). Additionally, the validity of the EA-SR-J was examined. Pearson’s correlation coefficients were calculated for each variable to explore the convergent validity of the EA-SR-J. The results showed that the EA-SR-J generally supported our hypothesis: higher Child Involvement, Affect Quality, and Mutual Attunement in the EA-SR-J were associated with mothers’ perceived emotional bonds with their children, higher attachment behaviors in children, and lower attachment style problems in mothers, whereas Hostility showed the opposite association. This aligns with previous studies that showed an association between EA and attachment [15,16,17]. In addition to the main validation analyses, exploratory findings revealed age-related differences in several EA-SR-J subscales. Specifically, Mutual Attunement and Affect Quality tended to decline with age, whereas Hostility and Child Involvement increased. Although these analyses were not central to the primary aims of this study, they suggest the potential developmental influence on parental emotional availability, which warrants further investigation.

Second, the mean total scores for each of the four subscales were compared to determine the discriminant validity of the EA-SR-J. As expected, mothers of children with ASD symptoms rated their EA-SR-J Child Involvement, Affect Quality, and Mutual Attunement significantly lower than mothers of children without ASD symptoms. They rated their children’s involvement behavior, emotional interaction, and understanding and attunement to each other’s intentions as challenging. This is consistent with previous studies showing that children with ASD are less involved and responsive in mother–child interactions than children with typical development [18,19]. In addition, mothers with depressive symptoms had significantly lower levels of Child Involvement, Affective Quality, and Mutual Attunement with their children and significantly higher levels of Hostility compared with mothers without depressive symptoms. These results support those of previous studies [10,20]. As described above, the EA-SR-J showed significant correlations with attachment and child-rearing styles, concepts similar to EA, and differences according to ASD and depressive symptoms. These results support the convergent and discriminant validity of the EA-SR-J.

The difference between the EA-SR-J and the original scale was that Intrusiveness was excluded from the former. In the original scale, Intrusiveness represented interference and overprotection by the mother. However, unlike in the original scale, Japanese mothers’ Intrusiveness in the EA-SR-J was perceived as a “good way of relating.” Only two of the six Intrusiveness items from the original scale were retained for this study. These items were included in the Affect Quality and Mutual Attunement factors and were interpreted positively (e.g., “When I’m at home together with my child, we interact and talk to each other a lot”). Japanese mothers possibly perceived the content of these items positively, rather than as representing intrusiveness and excessive interference. Kitayama and Miyamoto compared mother–child relationships in Japanese and Western cultures [30], and found that in Japanese culture, there is a strong sense of physical unity between the mother and child, such as sleeping together, which is a mutually cooperative practice that depends on the other party. In Western culture, mutually independent practices exist, such as sleeping in separate rooms. Such differences in the cultural background of the mother–child relationship may cause different ways of perceiving the EA-SR Intrusiveness factor, as answered by the mothers themselves. However, this study alone is insufficient to support this hypothesis, and it is necessary to examine the cultural backgrounds by targeting other countries in the future.

In addition, Intrusiveness is harder to recognize accurately than other factors. In a study of mothers with clinical depression, Vliegen et al. noted that it may be challenging to measure Intrusiveness using a conscious self-report questionnaire because it requires a high degree of reflection and self-awareness to realize that one’s involvement is intrusive for the child [10]. Notably, as rated by mothers, Intrusiveness on the EA-SR did not correlate significantly with Non-Intrusiveness on the EAS as rated by certifiers or accreditors [10]. Therefore, it is possible that mothers could not accurately assess their Intrusiveness, leading to its exclusion. The EAS is recommended over the EA-SR in clinical and other settings where circumstances permit [31]. The results of this study indicate that it may be preferable to use observational EAS to evaluate Intrusiveness.

Finally, we discuss the limitations of this study. First, as a measure of validity, future research should attempt to assess the correlation between observational EAS and EA-SR, which we did not do. As mentioned above, regarding the EA-SR-J’s Intrusiveness assessment, the EA-SR assessment only captures aspects perceived by mothers. In particular, it may be difficult to accurately monitor emotional relationships with children if mothers suffer from depression or other psychiatric disorders [10]. However, the EA-SR can be helpful in situations where assessment by observation is complex, or as an aid to observation to provide information on mothers’ perceptions of EA [32]. Therefore, considering the different perspectives of assessors, it is advisable to assess the relationship between the EA-SR-J and EAS. Second, retest reliability was not established. It is necessary to examine the degree of consistency when the same person is asked to complete the EA-SR-J multiple times at regular intervals. Third, the ASD symptoms and depressive tendencies used in this study to confirm the discriminant validity were based on scale cut-offs and scores, and not on clinical diagnoses; further studies involving clinical and non-clinical samples are needed. In sum, while the EA-SR-J demonstrated strong internal consistency and evidence of convergent and discriminant validity, its application to the Intrusiveness dimension remains complex. These complexities highlight the importance of combining self-report tools with observational methods in future research.

## 5. Conclusions

In this study, we translated and validated the EA-SR-J. The final scale consisted of 19 items across four factors, with good internal consistency (α = 0.81–0.88). Convergent validity was supported through correlations with emotional bonding, child attachment behaviors, maternal attachment styles, and parenting practices, while discriminant validity was confirmed via group comparisons based on children’s ASD symptoms and maternal depressive symptoms.

Although the Intrusiveness factor from the original scale was not retained, this finding may reflect cultural differences in parenting practices and the limitations of self-report measures. The EA-SR-J appears to be a reliable and valid tool for assessing mothers’ perceptions of EA in Japan.

These findings contribute to the growing body of research on EA by providing a culturally adapted tool for use in Japan, and underscore the importance of incorporating cross-cultural and multi-method approaches in future parenting research.

## Figures and Tables

**Table 1:** Demographic data of the participants (*N* = 739).

Characteristic		*n*	%
Mother’s work status	Company employee/Civil servant	215	(29.1)
	Temporary staff/Contract employees	28	(3.8)
	Part-time	122	(16.5)
	Student	0	(0.0)
	Housewife	346	(46.8)
	Unemployed	9	(1.2)
	Other	19	(2.6)
Number of cohabitants	1 person	9	(1.2)
	2 persons	15	(2.0)
	3 persons	300	(40.6)
	4 persons	272	(36.8)
	5 persons	88	(11.9)
	6 persons	42	(5.7)
	7 persons	9	(1.2)
	More than 8 persons	4	(0.5)
Spouse	Yes	705	(95.4)
	No	34	(4.6)
Birth order of children	first child	486	(65.8)
	2nd child	223	(30.2)
	3rd child	25	(3.4)
	4rd child	5	(0.7)
Child Affiliation	Nursery school	167	(22.6)
	Kindergarten	100	(13.5)
	childcare center	67	(9.1)
	Other	37	(5.0)
	Not attending preschool	368	(49.8)
Indication of developmental delay	Indicated	67	(9.1)
	Not indicated	672	(90.9)
Concerns about the child’s abilities and development	None at all	456	(61.7)
	Somewhat	244	(33.0)
	Very much	39	(5.3)
Concerns about the child’s behavior	None at all	425	(57.5)
	Somewhat	281	(38.0)
	Very much	33	(4.5)

Note. *N* = 739.

**Table 2:** Factor loadings and correlations of the EA-SR-J (19 items).

			EFA				CFA
Factor	Item No.		F1	F2	F3	F4	
F1: affect quality	1		0.83	0.12	0.02	−0.05	0.74
(α = 0.82/0.81)	3		0.75	0.07	−0.08	0.01	0.75
	2		0.75	−0.06	−0.01	−0.11	0.75
	7		0.67	−0.01	0.01	0.05	0.72
	14		0.53	−0.14	−0.01	0.15	0.66
	5		0.46	−0.12	0.14	0.01	0.35
F2: hostility	29		−0.06	0.93	−0.10	−0.04	0.91
(α = 0.87/0.88)	24		−0.02	0.75	0.05	0.03	0.82
	12		−0.09	0.73	0.04	−0.01	0.80
	6		0.11	0.70	0.10	0.06	0.71
F3: mutual attunement	21	R	0.16	−0.05	0.88	−0.02	0.70
(α = 0.81/0.82)	20	R	−0.12	−0.12	0.74	0.00	0.72
	10	R	0.02	0.18	0.56	0.14	0.70
	31	R	−0.05	0.08	0.56	−0.05	0.68
	16	R	−0.02	0.22	0.51	−0.08	0.66
F4: involvement	28		−0.10	−0.05	0.01	0.93	0.86
(α = 0.81/0.82)	27		−0.06	0.05	0.04	0.84	0.81
	19		0.22	0.04	0.01	0.50	0.62
	36		0.21	0.04	−0.13	0.47	0.64
Factor correlations				F1	F2	F3	F4
			F1	-	−0.39	−0.50	0.36
			F2	−0.29	-	0.69	0.25
			F3	−0.48	0.54	-	0.01
			F4	0.43	0.29	−0.10	-

Note. R = Reverse-coded items. Values of α are presented as EFA/CFA. Lower side of factor correlations is EFA data, upper side is CFA data.

**Table 3:** Pearson’s correlation coefficients between the EA-SR-J scores and other questionnaires.

			EA-SR-J							
	*M*	*SD*	Affect Quality		Hostility		Mutual Attunement		Involvement	
PBQ-J-14 ^a^	11.22	8.22	−0.55	**	0.58	**	−0.57	**	−0.15	*
ABCL ^b^										
Understanding of Caregiver’s Mind	26.79	4.62	0.32	**	0.03		0.19	**	0.50	**
Dysregulation of Emotions	24.54	6.66	−0.11	*	0.24	**	−0.48	**	−0.09	*
Secure Base	15.31	2.87	0.38	**	−0.02		0.15	**	0.45	**
ECR-GO ^c^										
Anxiety	57.14	17.64	−0.16	**	0.21	**	−0.43	**	−0.08	*
Avoidance	49.84	10.44	−0.18	**	0.09	*	−0.15	**	−0.04	
Childrearing Style ^d^										
Responsiveness	23.99	3.55	0.55	**	−0.31	**	0.35	**	0.30	**
Control	24.92	3.30	0.30	**	0.15	**	0.07		0.32	**

Note: ** *p* < 0.01, * *p* < 0.05. *M* = Average. *SD* = Standard deviation. ^a^ Postpartum Bonding Questionnaire—Japanese version of the 14-item scale. ^b^ Attachment Behavior Checklist. ^c^ Experiences in Close Relationships Inventory—Generalized Other version. ^d^ Maternal/Paternal Childrearing Style Scale.

**Table 4:** Comparison of EA-SR-J subscale scores between the ASD and non-ASD groups and maternal depression and non-depression groups.

	ASD ^a^	Non-ASD ^b^	*t*	*p*		*d*	95%CI		Depression ^c^	No Depression ^d^	*t*	*p*		*d*	95%CI	
	*n* = 46	*n* = 197					Lower	Upper	*n* =156	*n* = 583					Lower	Upper
Affect Quality	17.61	19.98	−3.29	0.002	**	−0.71	−1.04	−0.39	18.01	20.04	−5.53	0.000	**	−0.59	−0.77	−0.41
	(4.68)	(2.93)							(4.27)	(3.21)						
Hostility	7.74	7.47	0.42	0.677		0.07	−0.25	0.39	8.88	6.61	5.78	0.000	**	0.52	0.34	0.70
	(3.52)	(4.00)							(4.53)	(4.32)						
Mutual Attunement	10.72	12.87	−3.33	0.001	**	−0.55	−0.87	−0.22	9.91	13.50	−9.65	0.000	**	−0.87	−1.05	−0.69
	(4.22)	(3.88)							(4.31)	(4.07)						
Involvement	11.02	13.13	−4.55	0.000	**	−0.75	−1.07	−0.42	11.26	12.32	−3.30	0.001	**	−0.33	−0.51	−0.15
	(3.24)	(2.73)							(3.66)	(3.07)						

Note. ** *p* < 0.01. ^a,b^ M-CHAT score (3 or more items failed: ASD symptoms, 2 or fewer items failed: non-ASD symptoms). ^c,d^ PHQ-9 score (More than 10 points: depression symptoms, fewer than 9 points: no depression symptoms).

## Data Availability

The data presented in this paper are available on request from the corresponding author.
